# Supplementary material for: Tiled-ClickSeq for targeted sequencing of complete coronavirus genomes with simultaneous capture of RNA recombination and minority variants
Source: eLife. 2021 Sep 28;10:e68479. doi: 10.7554/eLife.68479 (PMC8478411; doi:10.7554/eLife.68479)
Supplement: Transparent reporting form [file elife-68479-transrepform1.docx]

***eLife’s* transparent reporting form**

We encourage authors to provide detailed information *within their submission* to facilitate the interpretation and replication of experiments. Authors can upload supporting documentation to indicate the use of appropriate reporting guidelines for health-related research (see [EQUATOR Network](http://www.equator-network.org/%20)), life science research (see the [BioSharing Information Resource](https://biosharing.org/)), or the [ARRIVE guidelines](http://www.plosbiology.org/article/info:doi/10.1371/journal.pbio.1000412) for reporting work involving animal research. Where applicable, authors should refer to any relevant reporting standards documents in this form.

If you have any questions, please consult our Journal Policies and/or contact us: [editorial@elifesciences.org](mailto:editorial@elifesciences.org).

**Sample-size estimation**

- You should state whether an appropriate sample size was computed when the study was being designed
- You should state the statistical method of sample size computation and any required assumptions
- If no explicit power analysis was used, you should describe how you decided what sample (replicate) size (number) to use

Please outline where this information can be found within the submission (e.g., sections or figure legends), or explain why this information doesn’t apply to your submission:

We do not compare populations or draw statistical conclusions about changes in groups in this manuscript, so this does not apply here.

**Replicates**

- You should report how often each experiment was performed
- You should include a definition of biological versus technical replication
- The data obtained should be provided and sufficient information should be provided to indicate the number of independent biological and/or technical replicates
- If you encountered any outliers, you should describe how these were handled
- Criteria for exclusion/inclusion of data should be clearly stated
- High-throughput sequence data should be uploaded before submission, with a private link for reviewers provided (these are available from both GEO and ArrayExpress)

Please outline where this information can be found within the submission (e.g., sections or figure legends), or explain why this information doesn’t apply to your submission:

This information is provided in the main text and methods section where applicable.

As this was a simple sequencing project, no data were excluded.

High-throughput data have been deposited and are publicly accessible in SRA, with the exception of raw sequencing data derived from clinical samples to protect PHI.

**Statistical reporting**

- Statistical analysis methods should be described and justified
- Raw data should be presented in figures whenever informative to do so (typically when N per group is less than 10)
- For each experiment, you should identify the statistical tests used, exact values of N, definitions of center, methods of multiple test correction, and dispersion and precision measures (e.g., mean, median, SD, SEM, confidence intervals; and, for the major substantive results, a measure of effect size (e.g., Pearson's r, Cohen's d)
- Report exact p-values wherever possible alongside the summary statistics and 95% confidence intervals. These should be reported for all key questions and not only when the p-value is less than 0.05.

Please outline where this information can be found within the submission (e.g., sections or figure legends), or explain why this information doesn’t apply to your submission:

We do not perform or provide statistical tests in this manuscript and so this does not apply here.

(For large datasets, or papers with a very large number of statistical tests, you may upload a single table file with tests, Ns, etc., with reference to sections in the manuscript.)

**Group allocation**

- Indicate how samples were allocated into experimental groups (in the case of clinical studies, please specify allocation to treatment method); if randomization was used, please also state if restricted randomization was applied
- Indicate if masking was used during group allocation, data collection and/or data analysis

Please outline where this information can be found within the submission (e.g., sections or figure legends), or explain why this information doesn’t apply to your submission:

Experimental groups are not assigned in this manuscript.

**Additional data files (“source data”)**

- We encourage you to upload relevant additional data files, such as numerical data that are represented as a graph in a figure, or as a summary table
- Where provided, these should be in the most useful format, and they can be uploaded as “Source data” files linked to a main figure or table
- Include model definition files including the full list of parameters used
- Include code used for data analysis (e.g., R, MatLab)
- Avoid stating that data files are “available upon request”

Please indicate the figures or tables for which source data files have been provided:

We have provide multiple supplementary data files. The annotations and computer scripts can be used to re-process raw data (SData 1 and 2) and SData 3-5 can be used to regenerated the figures presented in this manuscript using common graphing software.

**Supplementary Data 1**: Annotations and sequences of tiled-primers used in this manuscript are provided in BED format.

**Supplementary Data 2**: Batch scripts provided all computational tools and parameters used and python3 scripts used in this study are provided.

**Supplementary Data 3**: A summary of all Single-Nucleotide Variants (SNVs) detected for all samples sequenced in this study are provided. Each unique sample/isolate is listed, together with the SNVs relative to the WA-1 (NC_045512.2) strain in different NGS library preparation methods and sequencing platforms. The accession number for each reconstructed genome deposited in GenBank is also indicated.

**Figure 4-source data 1**: The frequency of all mapped nucleotides at each genome coordinate for each WRCEVA isolate is provided. The reference genome, nucleotide coordinate and expected reference Nucleotide is provided. Total read coverage and the numbers of each non-reference nucleotide are also shown. Finally, the mismatch/error rate at each site is provided which reveals minority variants in each isolate.

**Figure 7-source data 1**:BED files of RNA recombination events detected by ViReMa in the Tiled-ClickSeq data from each WRCEVA isolate and clinical sample.
